# Supplementary material for: Highly Sensitive Piezoresistive Pressure Sensor Based on Super-Elastic 3D Buckling Carbon Nanofibers for Human Physiological Signals’ Monitoring
Source: Nanomaterials (Basel). 2022 Jul 22;12(15):2522. doi: 10.3390/nano12152522 (PMC9330309; doi:10.3390/nano12152522)
Supplement: Supplementary file 1 [file nanomaterials-12-02522-s001.zip › nanomaterials-1828016-supplementary proof-revised.pdf]

nanomaterials

Supplementary Materials

Highly Sensitive Piezoresistive Pressure Sensor Based on Super-Elastic 3D Buckling Carbon Nanofibers for Human Physiological Signals' Monitoring

Zhoujun Pang [1,2], Yu Zhao [3], Ningqi Luo [3], Dihu Chen [1,*] and Min Chen [2]

[1] State Key Laboratory of Optoelectronic Materials and Technologies, School of Electronics and Information Technology, Sun Yat-sen University, Guangzhou 510275, China; pangzhj3@163.com
[2] School of Physics, Sun Yat-sen University, Guangzhou 510275, China; stscm@mail.sysu.edu.cn
[3] School of Materials and Energy, Guangdong University of Technology, Guangzhou 510006, China; zhaoyu@gdut.edu.cn (Y.Z.), nqluo@link.cuhk.edu.hk (N.L.)
* Correspondence: stscdh@mail.sysu.edu.cn

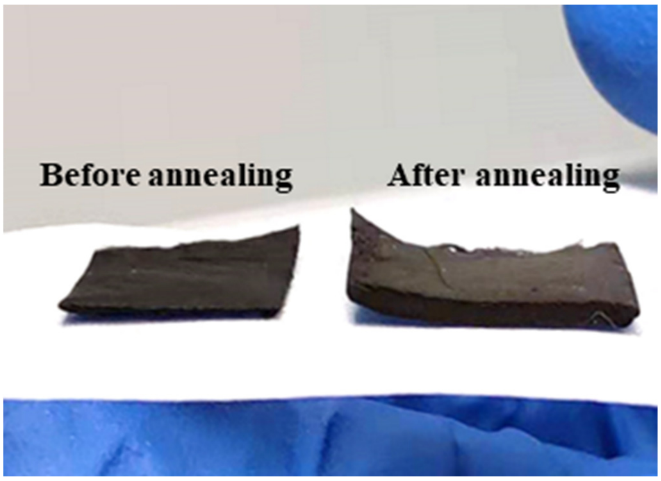

Figure S1. Photograph of the 3D BCNF before and after annealing.

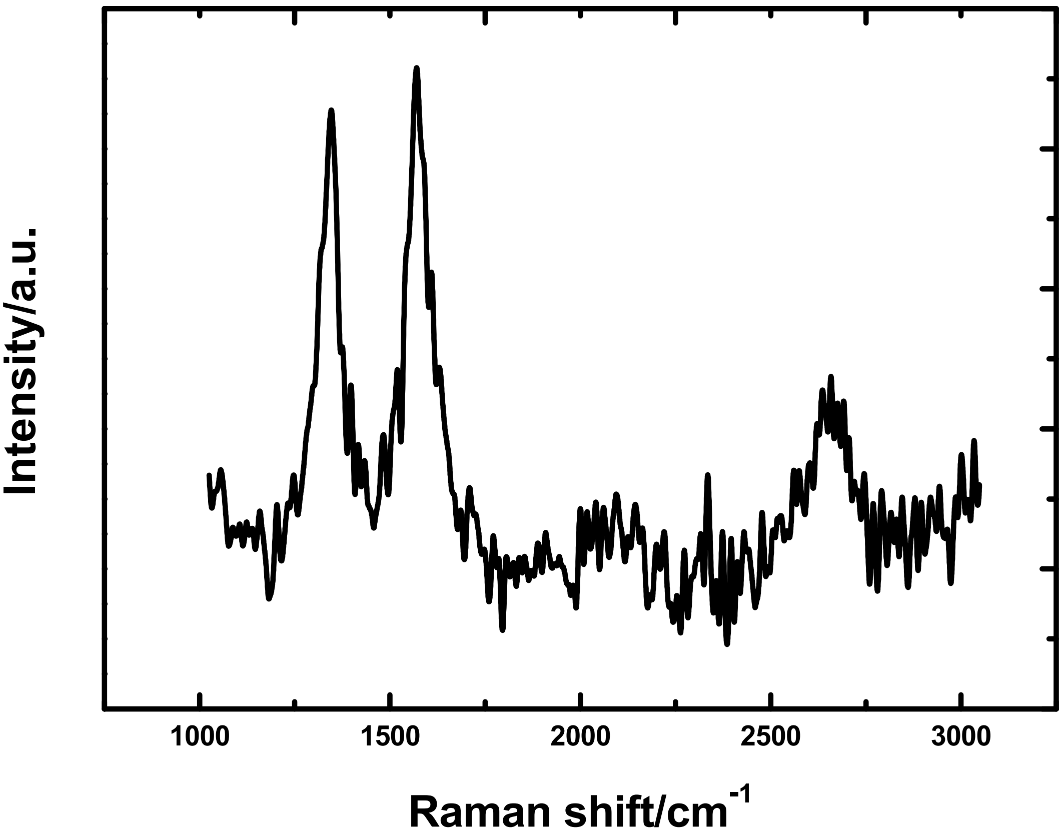

Figure S2. Raman spectra of 3D BCNF.

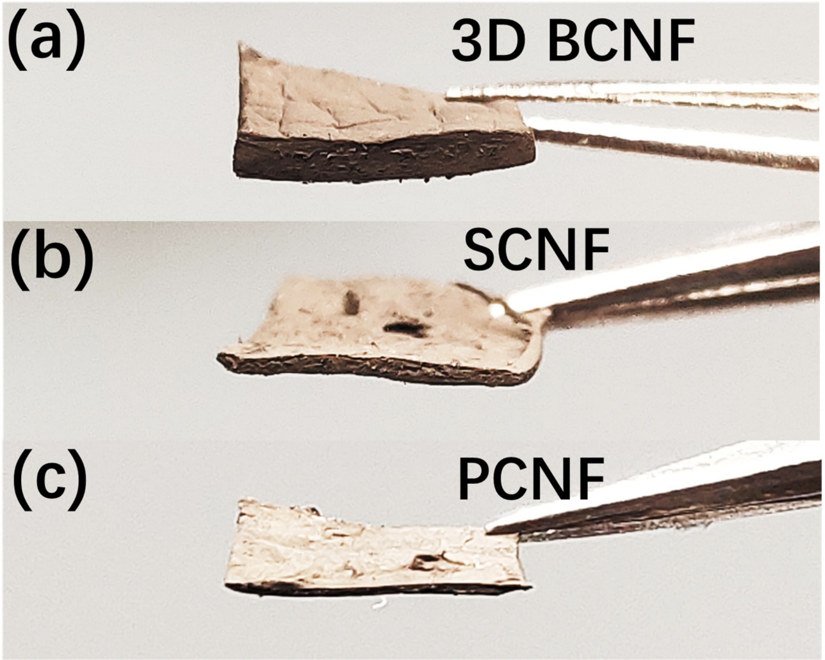

Figure S3. Photograph of (**a**) 3D BCNF, (**b**) SCNF, and (**c**) PCNF with different thicknesses after annealing.

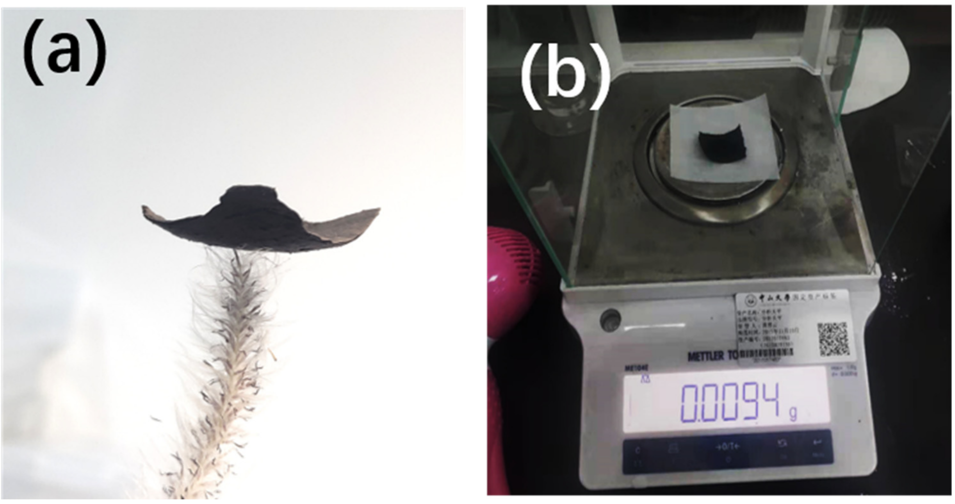

Figure S4. Photograph of 3D BCNF (**a**) standing on a soft hair of a dog's tail. (**b**) The weight displayed on an electronic balance.

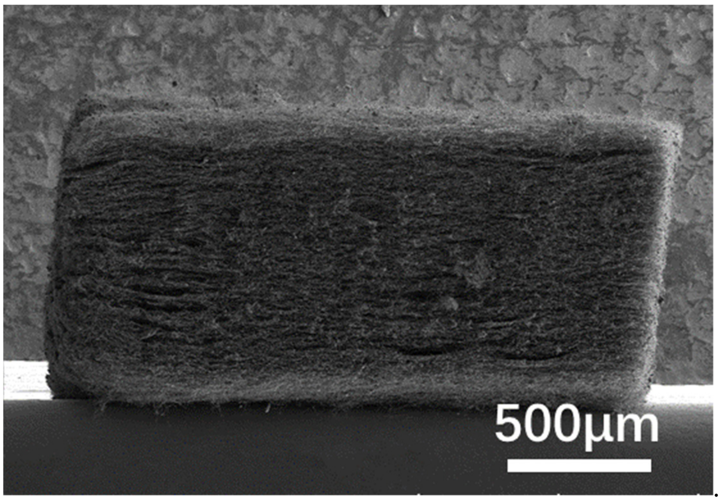

Figure S5. SEM image of the cross-section of 3D BCNF.

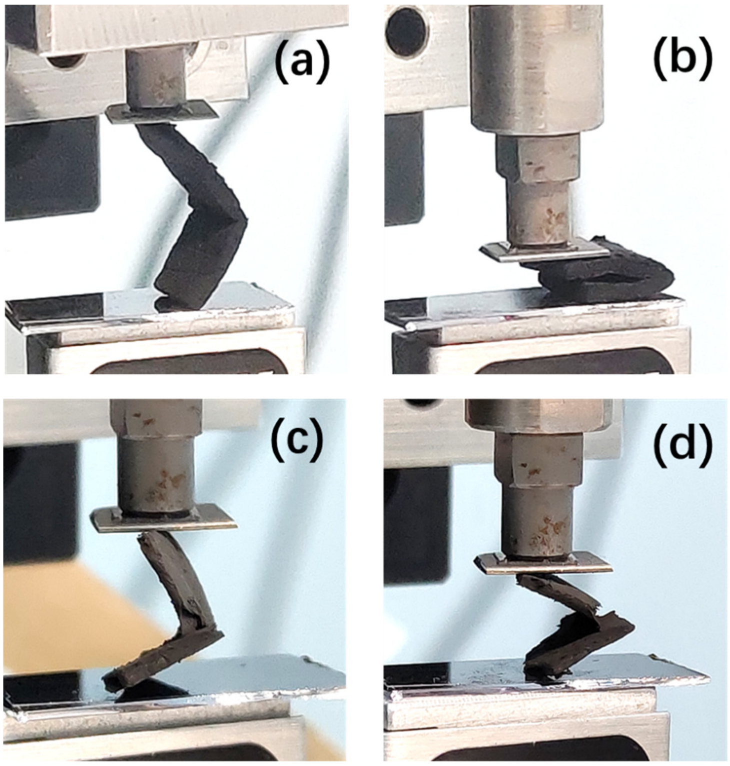

Figure S6. (**a**,**b**) 3D BCNF was bent at 180° during 10^3 cycles. (**c**,**d**) SCNF was fractured during the first cycle.

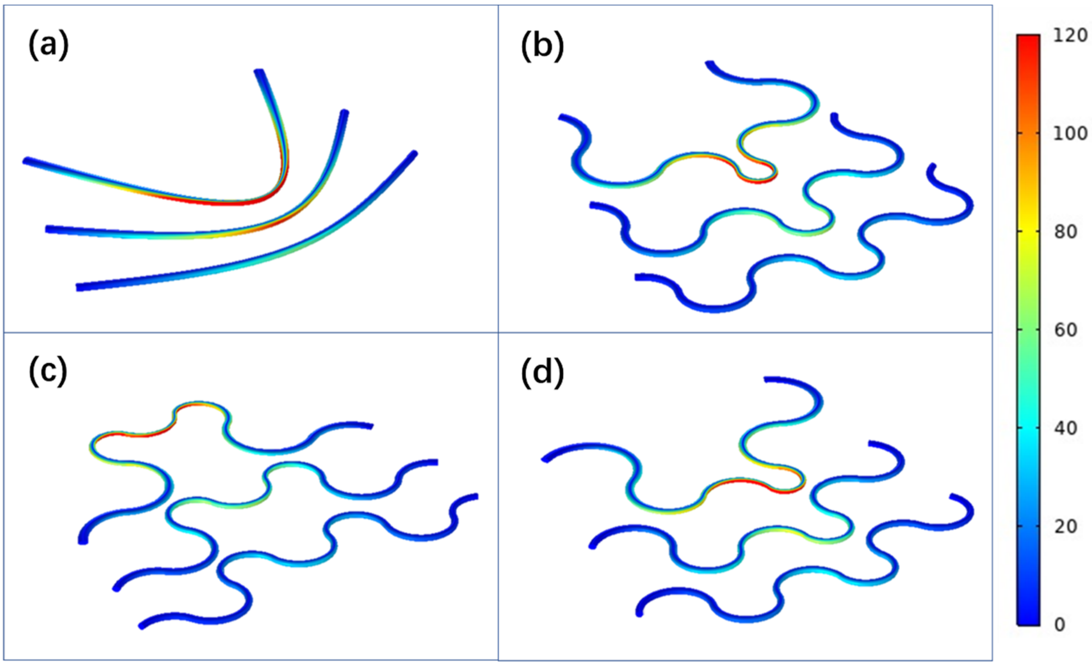

Figure S7. Mechanical simulations of (**a**) straight fiber, and serpentine spring fiber at (**b**) forward bending direction, (**c**) opposite bending directions, and (**d**) different positions.

Mechanical simulation: The large geometric deformation forces for different states of carbon fibers were investigated by simulating cylindric serpentine springs and straight fibers, using the finite element method.

The governing equations for the linear elastic deformation process of the material can be expressed as:

$$\nabla \cdot (C:\varepsilon)=0 \tag{S1}$$

$$\varepsilon = \frac{1}{2}[(\nabla L)^T + \nabla L] \tag{S2}$$

$$C = C(E,\nu) \tag{S3}$$

Here, C represents the fourth-order elastic tensor, E is Young's modulus, ν is Poisson's ratio, ε is the total stress, and L is the displacement.

The displacement loadings were applied to both ends of the fiber mat to produce bending. In the simulations, the geometric center of the interface of the model was a fixed boundary, and constant stress was applied in the y direction (of the left and right boundary) of the model. As a result, the applied stress can be formulated as:

$$(C:\varepsilon) = \begin{cases} 0 & x \\ const & y \\ 0 & z \end{cases} \tag{S4}$$

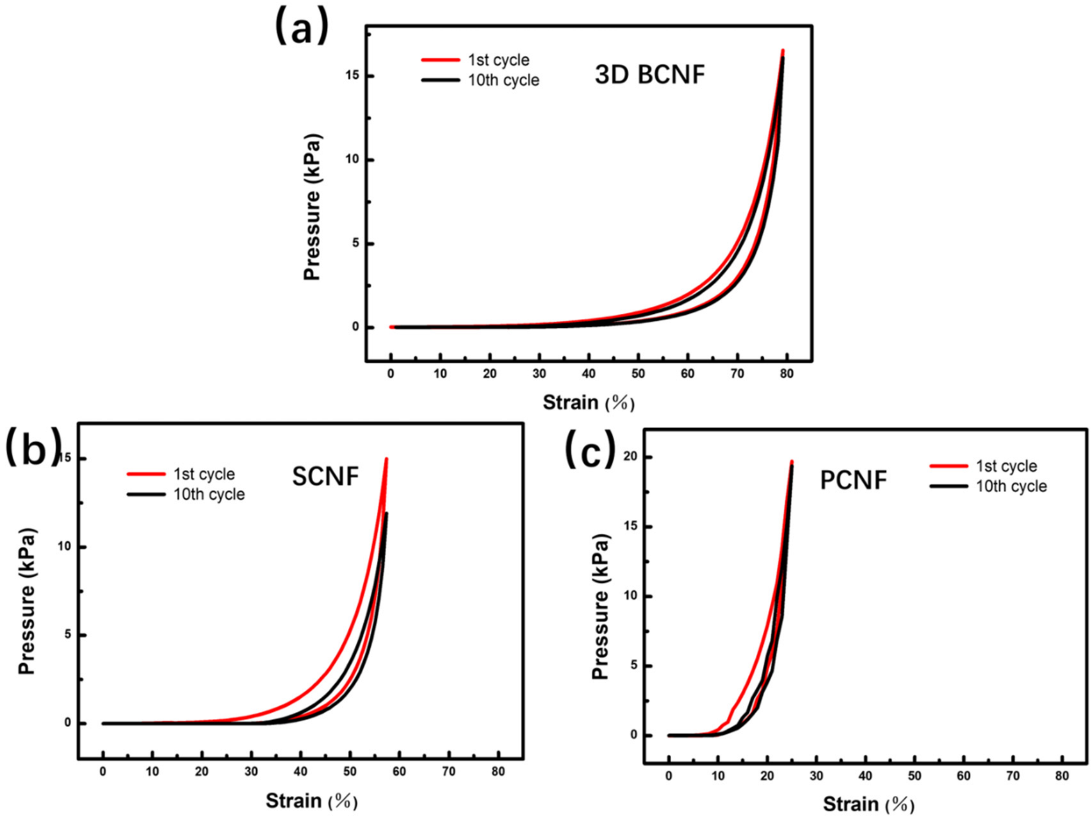

Figure S8. Stress–strain curves of compression of the (**a**) 3D BCNF, (**b**) SCNF, and (**c**) PCNF.

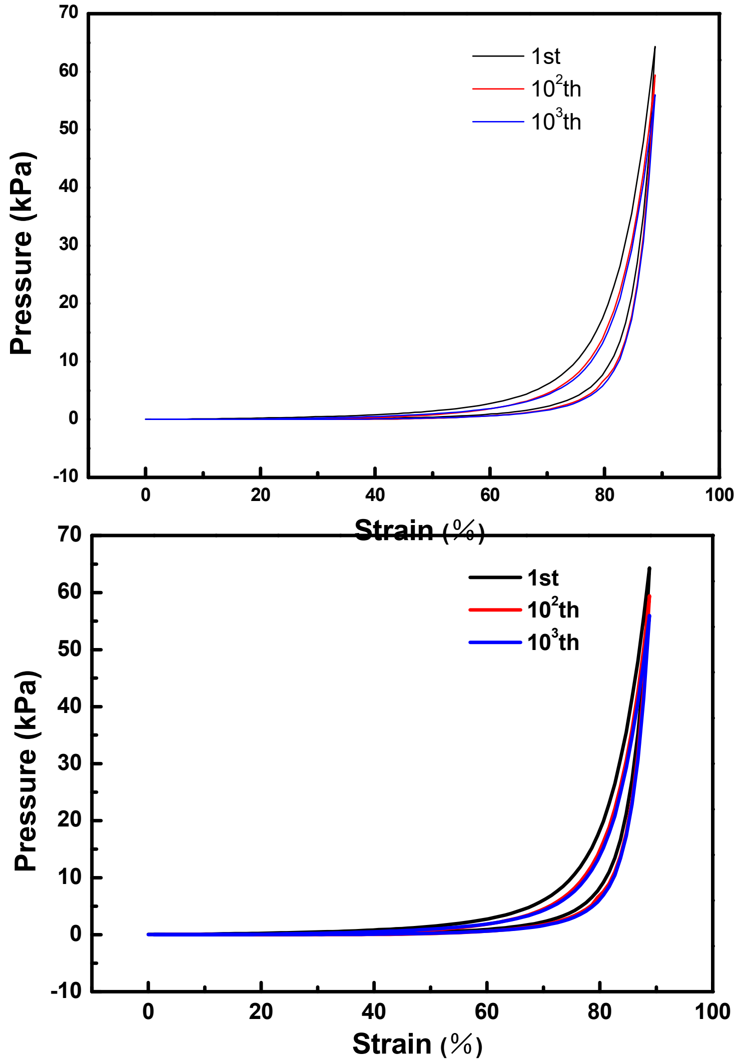

Figure S9. The stress–strain curves of 3D BCNF compressed for 10^3 cycles at a strain level of 90%.

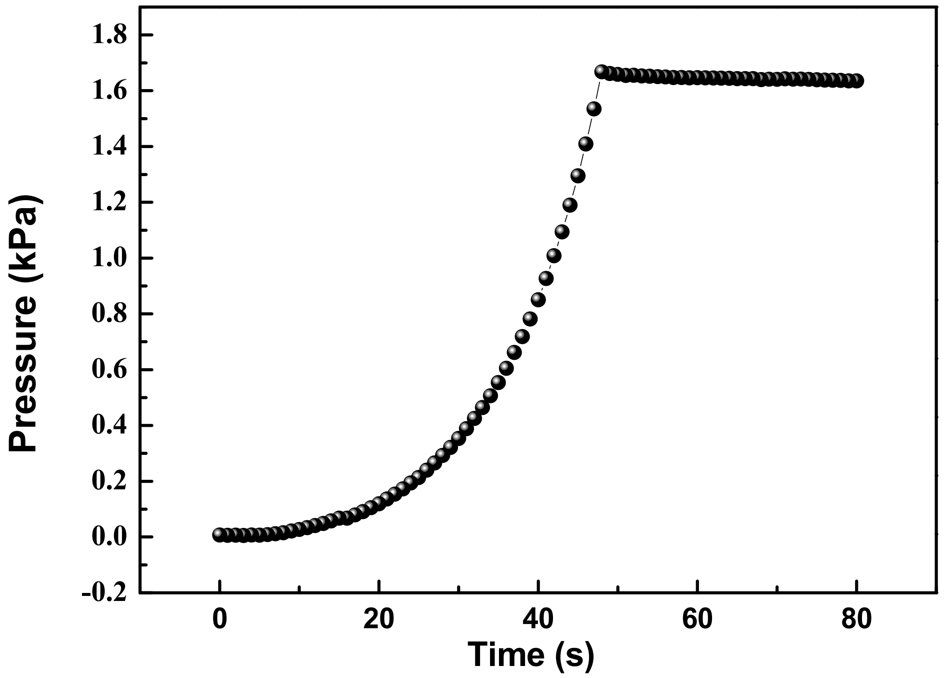

Figure S10. Stress–time curve in the static compression test.

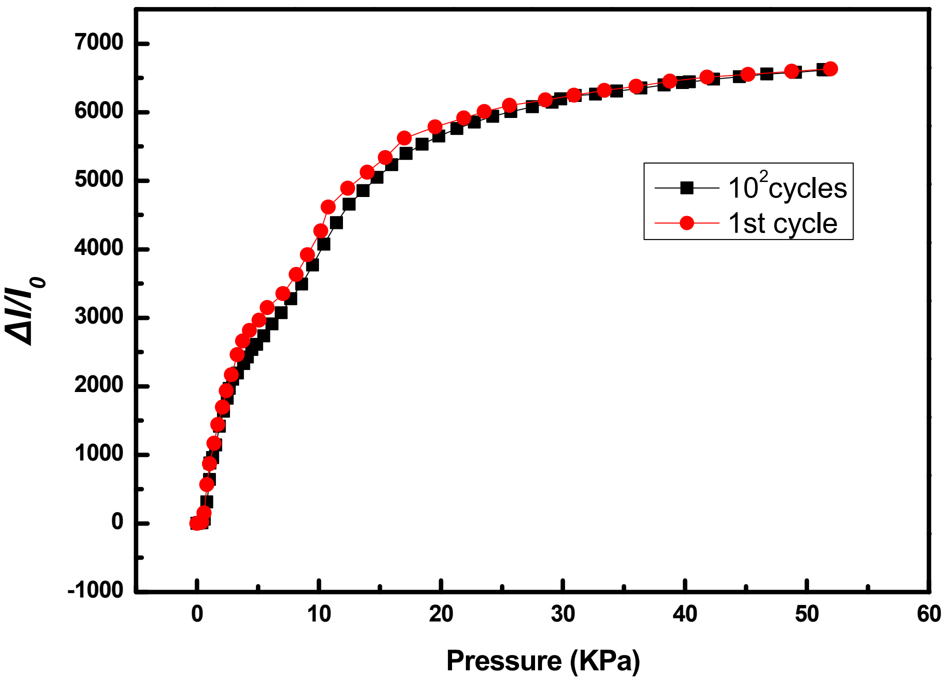

Figure S11. Repeat piezoresistive performance tests of the 3D BCNF sensor.

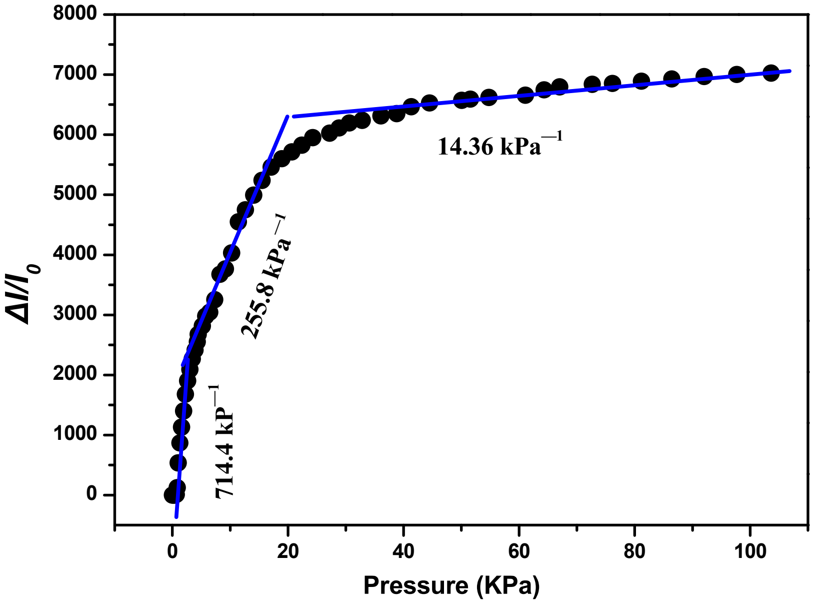

Figure S12. Pressure sensitivity curve of the 3D BCNF sensor at large pressure ranges.

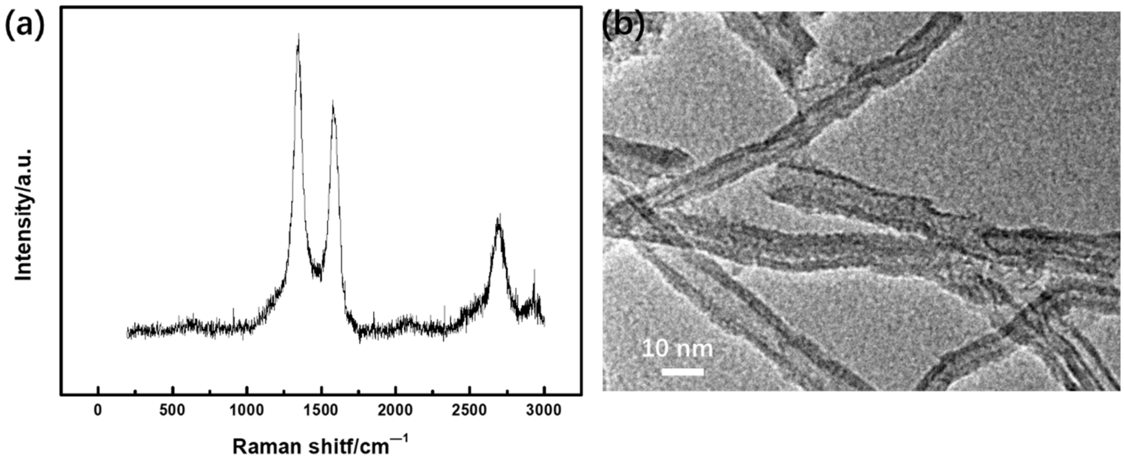

Figure S13. (**a**)Raman spectra and (**b**) TEM image of CNT.

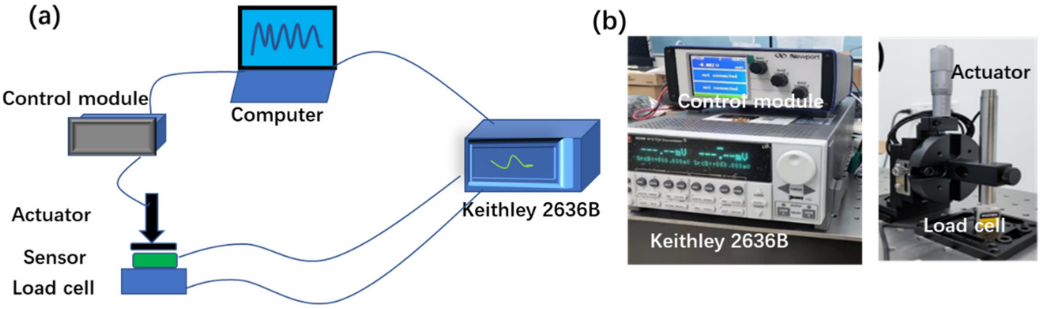

Figure S14. (**a**)The schematic diagram and (**b**) photos of the experimental setup.

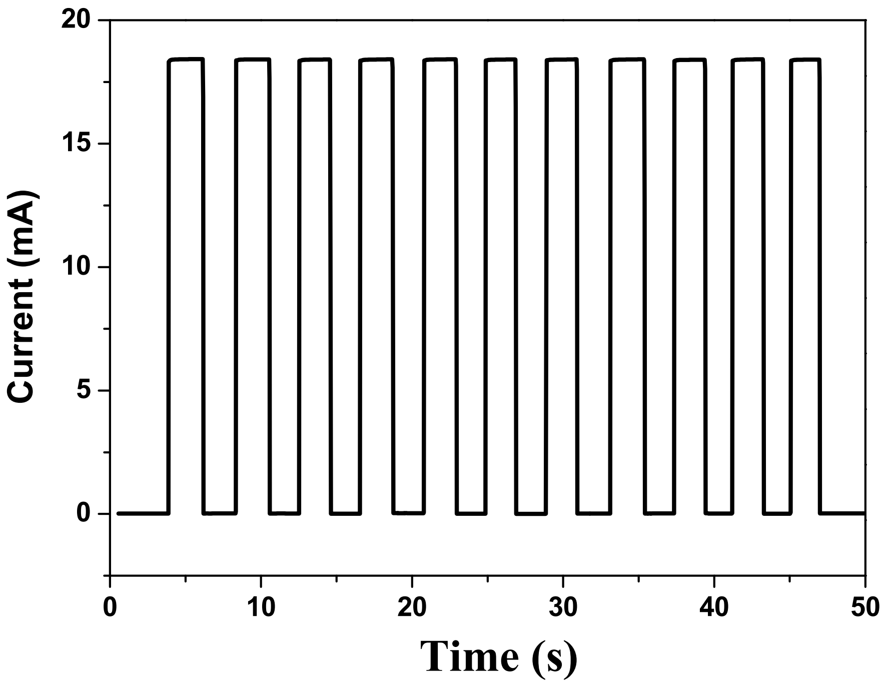

Figure S15. Cyclic pressure test at 75 kpa.

Table S1. Comparisons of mechanical performances of our 3D buckling carbon nanofibers with other carbon-based materials.

Material	Density (mg cm^{-3})	Speed of Rebound (mm s^{-1})	Reference
Graphene-coated CNT aerogels	14	11.2	15
Carbon nanofiber aerogels	9.2	860	20
3D carbon aerogels	18.3	400	22
Carbonaceous nanofibrous aerogels	5	1.3	30
3D graphene—PDMS	14.1	580	42
Graphene composite aerogel	5.1	117	49
3D buckling carbon nanofibers	25	950	This work

Table S2. Comparison of pressure sensitivity and other versatile properties of various 3D porous materials.

	Sensitivity (kPa^{-1})	Pressure range (kPa)	Response time (ms)	Reference
3D carbon nanofiber	1.41	4.5	300	38
3D graphene aerogel	28.62	14	37	27
3D carbon aerogels	114.6	10	189	23
Carbonaceous nanofibrous aerogels	1.02	5	100	52
3D graphene—PDMS	15.9	60	1.2	4
Graphene composite aerogel	609	10	232	24
Graphene/biomass aerogels	13.89	12	120	12
Carbon nanofiber aerogels	0.0057	50	-	20
PU@CNT	51.53	16	-	11
Wood-derived carbon aerogel	5.16	16.89	65	31
Composite aerogel	0.14	100	220	19
MX/rGO aerogel	22.56	3.5	245	26
3D buckling carbon nanofibers	714.4	120	23	This work
